# Spatial coding of ordinal information in short- and long-term memory

**DOI:** 10.3389/fnhum.2015.00008

**Published:** 2015-01-30

**Authors:** Véronique Ginsburg, Wim Gevers

**Affiliations:** Center for Research in Cognition and Neurosciences (CRCN), Faculté des Sciences Psychologiques et de l’Éducation, Action Bias & Control Group, Université Libre de Bruxelles (ULB)Brussels, Belgium

**Keywords:** numbers, space, SNARC effect, ordinal coding, working memory, long-term memory

## Abstract

The processing of numerical information induces a spatial response bias: Faster responses to small numbers with the left hand and faster responses to large numbers with the right hand. Most theories agree that long-term representations underlie this so called SNARC effect (Spatial Numerical Association of Response Codes; Dehaene et al., 1993). However, a spatial response bias was also observed with the activation of temporary position-space associations in working memory (ordinal position effect; van Dijck and Fias, 2011). Items belonging to the beginning of a memorized sequence are responded to faster with the left hand side while items at the end of the sequence are responded to faster with the right hand side. The theoretical possibility was put forward that the SNARC effect is an instance of the ordinal position effect, with the empirical consequence that the SNARC effect and the ordinal position effect cannot be observed simultaneously. In two experiments we falsify this claim by demonstrating that the SNARC effect and the ordinal position effect are not mutually exclusive. Consequently, this suggests that the SNARC effect and the ordinal position effect result from the activation of different representations. We conclude that spatial response biases can result from the activation of both pre-existing positions in long-term memory and from temporary space associations in working memory at the same time.

## Introduction

In the domain of numerical cognition it is well known that the processing of numbers and space is highly related. The first scientific articles, illustrating this link, date back to the 19^th^ century. Galton published two papers in which he reported that some persons explicitly represent numbers in a spatial organized way, that he termed “number form” (Galton, 1880a,b). One of the most striking demonstrations of such a numbers-space association is the Spatial Numerical Association of Response Codes (SNARC) effect (Dehaene et al., 1993). This effect reveals an association between numerical magnitude and lateralized motor responses: participants respond faster to small numbers with the left hand side and to large numbers with the right hand side. The SNARC effect has been observed in multiple studies using different design, tasks settings and populations (see Wood et al., 2008). One of these tasks is the parity judgment task (Dehaene et al., 1993). In this task, participants have to classify target numbers as odd or even by pressing a left or a right response button. The SNARC effect is also observed when magnitude information has to be accessed more explicitly such as in a magnitude comparison task (Dehaene et al., 1990). In this task, participants have to judge whether a target number is smaller or larger than a reference number (for instance: 5) by pressing a left or a right-sided response button.

Different frameworks exist interpreting the SNARC effect: Some argue that the SNARC effect results from an association between the position of the number on a left-to-right (or right-to-left depending on reading habits) oriented mental number line (MNL) and the position of the response (Restle, 1970; Dehaene et al., 1993). Other researchers argue that the SNARC effect results from associations between magnitude concepts such as “small”, or “large” and spatial concepts such as “left” or “right”. For instance Proctor and Cho (2006) suggest that stimulus and response characteristics are coded as positive and negative polarities. The SNARC effect then results from the fact that small and left are coded as negative polarity and large and right as positive polarity. Similarly, Gevers et al. (2006) proposed a computational model that explains the SNARC effect assuming associations between concepts such as small-large and left-right. Despite the differences in interpretation of the SNARC effect, all of these accounts converge on the idea that long-term representations underlie the interactions between numbers and the side of response.

However, the association between numbers and space is more flexible than one could expect on the basis of such long-term associations. Early reports on the SNARC effect demonstrated that relative instead of absolute magnitude information is associated with response side (Dehaene et al., 1993; Fias et al., 1996). For example, the number 5 was responded to faster with the left hand when it was relatively small within the range (e.g., numbers ranged from 4 to 9), but the same number was responded to faster with the right hand when it was relatively large within the range (e.g., from 1 to 5). More recently, Ben Nathan et al. (2009) investigated deeper the range effect in a magnitude comparison task by changing the standard reference from trial to trial. They also observed that the SNARC effect was influenced by the relative instead of the absolute magnitude of digits. For example, the number “3” was associated with the left hand when the referent was “4”, but with the right hand when the referent was “2”. Additionally, Bächtold et al. (1998) demonstrated that the SNARC effect could be reversed by means of mental imagery. When subjects were asked to imagine numbers on a clock face, the SNARC effect reversed, because now small numbers occurred on the right side of the clock face and large numbers on the left side. Similarly, Shaki and Fischer (2008) showed that Russian-Hebrew bilinguals presented a normal SNARC effect if they had to read a Russian text (reading from left to right) just before the SNARC task but this effect was significantly reduced if they had to read a Hebrew text (reading from right to left) just before.

This high flexibility of the association between numbers and response side seems to indicate that number-space associations are built up during the task. This suggests that working memory could play an important role in the creation of these associations (e.g., Fias et al., 2011). In agreement with this view, several studies demonstrated that the availability of working memory resources is necessary, under certain circumstances, to observe number-space associations (Herrera et al., 2008; van Dijck et al., 2009). Following these observations, van Dijck and Fias (2011) more directly investigated the role of working memory in the creation of number-space associations. In their experiment, participants were asked to keep a sequence of five numbers in working memory (randomly chosen between 1 and 10). Subsequently, participants had to perform a parity judgment task, but only on numbers that belonged to the memorized sequence. This go-nogo procedure was used to ensure that the numbers had to be retrieved from working memory. Interestingly, using this paradigm, the researchers observed that lateralized responses were not associated with the magnitude of the numbers (no SNARC effect) but with the ordinal position of the numbers in the memorized sequence. Regardless of their magnitude, numbers from the beginning of the memorized sequence were responded to faster with the left hand side whereas numbers at the end of the sequence were responded to faster with the right hand side (e.g., from here on this observation will be termed the ordinal position effect). On the basis of this observation, van Dijck and Fias (2011) proposed a working memory account of the SNARC effect as an alternative to the long-term representation of numbers (van Dijck and Fias, 2011). It was more specifically suggested that the SNARC effect observed in typical situations (e.g., magnitude comparison, parity judgment) does not result from a long-term representation of numbers but rather from the creation of a task relevant ordinal sequence in working memory. This interpretation is in accordance with the fact that a SNARC-like effect also appears with non numerical stimuli with an ordinal structure such as letters of the alphabet (Gevers et al., 2003), overlearned new sequences (Van Opstal et al., 2009; Previtali et al., 2010) or musical tones (Lidji et al., 2007).

It is well known that working memory is not a unitary process. Most models on working memory functioning make a distinction between maintaining and retrieving information from working memory (Baddeley and Hitch, 1974; Oberauer, 2002). In a follow up study, Ginsburg et al. (2014) examined whether and how the type of processing (maintenance or retrieval) influenced the mapping between numbers and lateralized responses. They replicated the observations of van Dijck and Fias (2011): an ordinal position effect was observed when participants needed to retrieve memorized numbers during the classification phase (go-nogo procedure). In another task, the go-nogo procedure was removed from the design. Participants had to respond to all digits, both inside and outside the working memory sequence (respond-all procedure). This way, the memorized numbers were maintained for later recall while retrieval was not required during the classification of the numbers. Using this respond-all procedure the ordinal position effect was no longer observed while the SNARC effect remerged. These observations were explained by referring to the working memory model proposed by Oberauer (2002). In this model, a distinction is made between items that need to be maintained for later recall and items that need to be retrieved while being maintained. Maintained items would be represented as increased activations in long-term memory. The retrieval of these items, maintained in working memory, would result in the creation of new temporary bindings between these items. Oberauer (2010) suggested that these temporary bindings link the items with locations in mental space such as the ordinal position in a list. On the basis of this framework, Ginsburg et al. (2014) speculated that the SNARC effect would typically result from increased activations in long-term memory while the ordinal position effect results from the new bindings that are created between the retrieved items.

The current study was set up to investigate the relation between the SNARC effect and the ordinal position effect. In the theoretical working memory framework of van Dijck and Fias (2011), it was hypothesized that both effects are derived from temporary position-space associations. Following this hypothesis, the association between numbers and space is not long-term but created during task performance. For instance, when participants perform a simple numerical classification task (e.g., magnitude comparison task), the sequence of numbers is strategically activated in its canonical order to facilitate task execution (van Dijck et al., 2013). When, on the other hand, participants retrieve numbers from a newly memorized sequence (go-nogo procedure), it would be this new relevant sequence that is activated to perform the task. In other words, depending on the specific task-set, only one sequence is preferentially activated in working memory.

According to an alternative theoretical proposal (Ginsburg et al., 2014), the canonical representation of the numbers is automatically activated, whenever a numerical task has to be performed, regardless of the task-set. However, when a new memorized sequence becomes relevant to perform the task, an imbalance is created between the activation of the irrelevant canonical representation and the activation of the new relevant sequence. A natural consequence of this view is that the SNARC effect and the ordinal position effect could be observed simultaneously provided that a balance exists in the activation of both short- and long-term sequences. As stimulus material, numbers are ideal to investigate this point because they can easily be used as material in working memory while at the same time continue to be overlearned in long-term memory.

## Experiment 1

According to the framework of Ginsburg et al. (2014), it can be argued that the SNARC effect is not observed in the go-nogo task used by van Dijck and Fias (2011) because there is an imbalance between the activation of the numerical long-term representations and the new memorized sequence. As suggested by Oberauer (2010, on p. 281): “*the more active a representation, the easier it is to retrieve it*”. So, the new memorized sequence should be more activated than the canonical representation because this information must be retrieved to perform the task correctly. In this experiment, to increase the activation of numerical long-term representations, participants were asked to respond to all numbers (inducer task: respond all paradigm) before performing the diagnostic go-nogo task.

### Materials and methods

#### Participants

In total, 42 paid volunteers (on average 22.00 years (SD = 2.41); 25 females (four left handed) and 17 males (two left handed)) participated in this experiment. All participants were undergraduate students recruited via an announcement on Facebook. Participants received 10 euros as compensation for their participation. The ethical committee approved this study and participants received a debriefing after completing a single 60 min session. All participants were naive with respect to the purpose of the experiment.

#### Material

The experiment was performed using E-Prime 2 Professional Software (Psychology Software Tools). Participants were seated in a quiet room approximately 50 cm from a 17 inch LCD computer screen with a resolution of 1280 × 1024 pixels. The motor responses were collected via button presses on a response box. Each digit (approximately 1.37°) was presented on the computer screen in white color on a black background.

#### Stimuli and procedure

The experiment consisted of 40 different blocks: 20 inducer blocks and 20 diagnostic blocks. Two response mappings were introduced, a SNARC compatible response mapping (small numbers, left response—large numbers, right response) and a SNARC incompatible response mapping (small numbers, right response—large numbers, left response). Each participant started with 10 inducer blocks followed by 10 diagnostic blocks. After this, the response mapping was switched and the participant again performed 10 inducer followed by 10 diagnostic blocks. Which response mapping was performed first was counterbalanced across participants, but the same order was maintained in the first and the second half of the experiment.

Each block was divided in three subsequent phases: an encoding phase, a classification phase, and a control phase. During the encoding phase, five digits (randomly chosen in the range from 1 to 10) were successively presented during 1500 ms at the center of the screen (with an interval of 200 ms). Each digit could be presented only once in a sequence. The first digit of this sequence was preceded by a fixation cross during 500 ms. Participants were instructed to memorize this numerical sequence in the correct order.

During the classification phase, participants continued to keep the memorized numerical sequence in mind while performing a magnitude comparison task. Participants had to indicate whether the presented target number was small (range 1–5) or large (range 6–10). In each block, all numbers ranging from 1 to 10 were randomly presented twice with the restriction that the same number could not be repeated on consecutive trials. A trial consisted of a fixation cross (500 ms) followed by a target number. The response deadline was set to 1500 ms. After this deadline or after a response, the next trial was initiated, following an inter-trial interval of 1000 ms. During the inducer blocks participants had to perform the magnitude comparison task on every presented number (respond all paradigm). During the diagnostic blocks participants continued with the magnitude comparison task but now responded only to numbers that belonged to the memorized sequence (go-nogo paradigm).

During the last phase, the control phase, participants had to judge by pressing on a response button whether or not a new sequence of five digits (sequentially presented in the center of the screen during 1000 ms with an interval inter-stimuli of 200 ms) was the same sequence as the one kept in memory.

The non-corresponding sequences were composed by the same five numbers of the memorized sequence but, at a random location, the order between two adjacent numbers was reversed. This phase is important to assure that participants memorized correctly the numerical sequence during the classification phase. For this reason, if the participant responded erroneously to the control phase during the diagnostic task, the entire block was introduced again at the end of the experiment. To ensure that all participants performed exactly the same inducer task, we decided to not repeat a block of trials with an incorrect response to the control phase. Rather, participants were eliminated from analyses if they made more than three errors to memorized blocks in the control phase.

After each block participants had the opportunity to take a break and started the next block by pressing a response button. Concerning the training, before each mapping condition (20 blocks each), participants performed one block with the respond all procedure and one block with the go-nogo procedure to get used to the task.

### Result and discussion

#### Inducer task—respond all procedure

The data of one participant were removed from the analysis because he made more than three errors during the control phase. For the analyses, we took into account only blocks with an accurate control phase and correct trials with reaction times (RTs) larger than 250 ms. Only 0.1% of data points were discarded with this RT cutoff.

**Figure 1:**
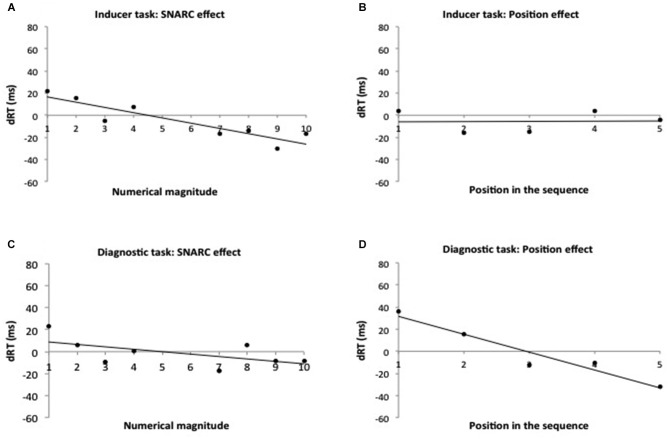
**Experiment 1: the SNARC effect and the ordinal position effect in the inducer and the diagnostic tasks**. Observed data and regression line of Experiment 1, representing RT differences between right and left hand responses in function of the numerical magnitude (in the inducer task **(A)**—in the diagnostic task **(C)** and in function of the ordinal position of digits in working memory (in the inducer task **(B)**—in the diagnostic task **(D)**. Positive values reflect faster left than right responses.

During the inducer task, participants performed correctly on average 8.7 blocks (SD = 1.38) on 10 blocks. During the magnitude comparison task, the average reaction time was 530.97 ms (SD = 72.91 ms) and the average number of errors was 3.65% (SD = 3.59). A sharp drop in performance was observed for the numbers 5 and 6 (mean of errors = 10.29%) compared to the other numbers (mean of errors = 1.99%). Classifying the numbers 5 and 6 as small or large seem particularly difficult because these numbers lie at the boundary of small and large categorizations. For this reason these numbers were removed from the analyses (e.g., Ginsburg et al., 2014). Nevertheless, a separate analysis with the numbers 5 and 6 included resulted in exactly the same pattern of results. Because in the inducer task participants responded to all numbers, a SNARC effect but no ordinal position effect was expected. To investigate the presence of this effect we used a repeated measure ANOVA with numerical magnitude (2: small numbers 1–4, large numbers 7–10) and response side (2: left, right) as within-subjects factors (recommended by Schwarz and Keus, 2004). No main effect was significant. However a SNARC effect was observed, indicated by a significant interaction between magnitude and response side (*F*_(1,40)_ = 4.44, *p* < 0.05, $\eta_{p}^{2}$ = 0.08). Participants responded faster to small digits with the left-hand side (mean RT = 506.34 ms, SD = 11.17) than with the right-hand side (mean RT = 516.03 ms, SD = 11.96) while they responded faster to large digits with the right-hand side (mean RT = 506.34 ms, SD = 11.17) than with the left-hand side (mean RT = 524.55 ms, SD = 11.80). A separate ANOVA was conducted to investigate the presence of the ordinal position effect for the memorized numbers with ordinal position (5: from 1 to 5) and response side (2: left, right) as within-subjects factors. No ordinal position effect was observed (*p* = 0.35). These analyses were complemented with a regression approach described by Lorch and Myers (1990; see also Fias et al., 1996). This method consists of computing the difference in RTs (dRT; RT right hand minus RT left hand) for each number (from 1 to 10, except 5 and 6) or for each position in working memory (from 1 to 5) separately. Per subject, these values were entered in a regression analysis with number or position as predictor. A *t-test* was performed to evaluate whether the regression weights of the group deviated significantly from zero. The regression analysis confirmed the presence of the SNARC effect (dRT= −6.49 ms, *t*_(40)_ = −2.16, *p* < 0.05) (Figure 1A) but no evidence was found for the presence of the ordinal position effect (*t*_(40)_= 0.06, *p*= 0.95) (Figure 1B).

In sum, replicating previous work (Ginsburg et al., 2014), when information inside working memory is maintained but not retrieved during the classification, a SNARC effect but no ordinal position effect was observed. The question now becomes whether this pre-activation of the numerical canonical order in the inducer task has an effect on the presence of the SNARC effect in the diagnostic task.

#### Diagnostic task—go-nogo procedure

The same cut-off criteria were maintained during the diagnostic task as during the inducer task. No data points were discarded with the RT cutoff of 250 ms. During this task, participants made on average a bit more than one error to the control phase across 10 blocks (for both conditions: odd-left/even-right and odd-right/even-left). As such, participants required on average 11.15 blocks (SD = 1.90) to finish the task having performed 10 correct sequences. During the magnitude comparison task, the average reaction time was 696.43 ms (SD = 98.43 ms) and the average number of errors was 4.85% (SD = 2.21). For the same reasons as in the inducer task, analyses were performed on all numbers except the numbers 5 and 6. Again, as in the inducer blocks, separate analyses on the entire range of numbers showed the same results. During this task, participants classified numbers as small or large only if they belonged to the memorized sequence. Because all responded stimuli were inside the sequence, we used repeated measures ANOVA with numerical magnitude (2: small, 1–4; large, 7–10), ordinal position (5: from 1 to 5) and response side (2: left, right) as within-subjects factors to investigate the presence of the SNARC effect and the ordinal position effect for the elements inside working memory. A main effect of position was observed (*F*_(4,160)_ = 12.28, *p* < 0.001, $\eta_{p}^{2}$ = 0.23). Average RTs per position increased gradually (652, 676, 681, 708, 705 for each position, respectively). A polynomial contrast confirmed the linear trends of these RTs (*F*_(1,40)_ = 37.00, *p* < 0.001, $\eta_{p}^{2}$ = 0.48), suggesting a serial search strategy. No other main effect reached significance. Indicating the presence of the ordinal position effect, a significant interaction was observed between ordinal position and response side (*F*_(4,160)_ = 4.89, *p* < 0.005, $\eta_{p}^{2}$ = 0.11). The interaction between numerical magnitude and response side, representing the SNARC effect, was not significant (*F*_(1,40)_ = 0.28, *p* = 0.60, $\eta_{p}^{2}$ = 0.01). The triple interaction between numerical magnitude, ordinal position, and response side was also not significant (*F*_(4,160)_ = 0.55, *p* = 0.70, $\eta_{p}^{2}$ = 0.01). The data were re-analyzed using a Lorch and Myers (1990) regression analysis. The dRTs decreased 16.18 ms per position (*t*_(40)_ = −3.82, *p* < 0.001) (Figure 1D). This was not the case for the SNARC effect, where the slope did not differ from zero (*t*_(40)_ = −0.75, *p* = 0.46) (Figure 1C).

**Figure 2:**
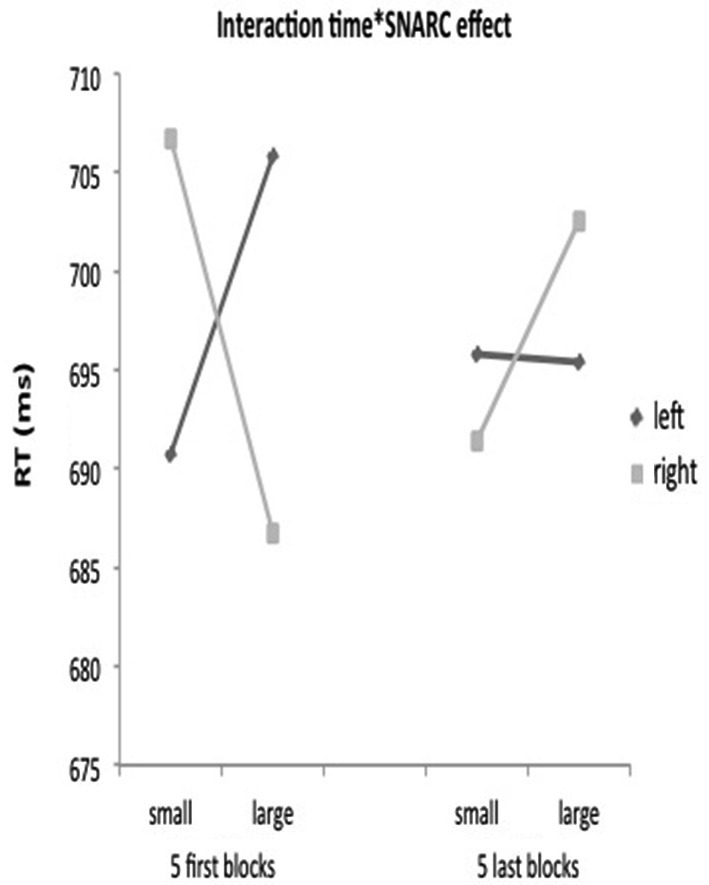
The interaction between time*magnitude*response in the diagnostic task of Experiment 1. Mean reaction times in the magnitude comparison task of the diagnostic part (Experiment 1) as function of the factors number magnitude (small–large), response side (left–right) and time (5 first blocks–5 last blocks).

Only an ordinal position effect was observed and no SNARC effect in the diagnostic task. Because the inducer task was presented blocked-wise before the diagnostic task, it is possible that its influence was limited to the first part of the diagnostic task. To investigate this possibility, the possible presence of the SNARC effect was investigated taking the time course of the diagnostic task into account. To this end, a repeated measures ANOVA was run with magnitude (2: < 5 or > 6), response side (2: left hand–right hand) and time (2: first five correct blocks, five last correct blocks) as within-subjects factors. This analysis revealed an interaction between time, numerical magnitude and response side (*F*_(1,40)_ = 4.20, *p* < 0.05, $\eta_{p}^{2}$ = 0.10) (Figure 2). Even though the interaction was significant, planned comparisons indicated no significant SNARC effect, neither for the first (*F*_(1,40)_ = 2.07, *p* = 0.16), nor for the second half (*F*_(1,40)_ = 0.27, *p* = 0.61).

A final repeated measures ANOVA with ordinal position (2: early, late), response side (2: left, right) and time (2: first five correct blocks, five last correct blocks) revealed a main effect of ordinal position (*F*_(4, 160)_ = 15.53, *p* < 0.001, $\eta_{p}^{2}$ = 0.28) and an interaction between position and response side (*F*_(4,160)_ = 4.52, *p* < 0.005, $\eta_{p}^{2}$ = 0.10) but no interaction of the ordinal position effect with time (*p* = 0.675).

As in previous studies (van Dijck and Fias, 2011; Ginsburg et al., 2014), when a go-nogo procedure was used, only an ordinal position effect but no SNARC effect was observed. Even though a SNARC effect was observed during the inducer phase, this seemed not to have a strong influence on the activation of the numerical canonical order during the diagnostic task. However, an exploratory analysis taking the influence of the time course of the diagnostic task into account revealed a triple interaction between magnitude, time and response side. This interaction suggests that the inducer task has an influence on the performance in the diagnostic task, but that this influence is limited in time.

## Experiment 2

In Experiment 2, the possible co-occurrence of the SNARC effect and the ordinal position effect is further explored. Even though based on exploratory analyses, the results of the previous experiment suggest that the inducer task has a time limited influence on the SNARC effect in the diagnostic task. This time, a task-switching paradigm is used to ensure that participants activate the numerical canonical order throughout the entire experiment. To this end, participants switched randomly between respond-all and go-nogo blocks.

An extra advantage of the task-switching paradigm is that it enables to investigate whether the spatial associations causing the ordinal position effect are created during encoding or during retrieval. On each block, an instruction was given with the written words “IN” or “ALL” to inform participants whether they had to perform the go-nogo paradigm (IN) or the respond all paradigm (ALL). This instruction was presented directly before or directly after the encoding phase. The process of encoding can be qualitatively different if participants know beforehand what task to perform on the encoded information compared to the situation where this information is given only after encoding. Therefore, if type of encoding is important for the ordinal position effect, this manipulation could have an influence on the associations between lateralized responses and ordinal position in working memory. If on the other hand, the spatial associations responsible for the ordinal position effect are created during retrieval, no such difference is expected.

### Materials and methods

#### Participants

In total, 52 paid volunteers (on average 22.19 years (SD = 2.33); 39 females (4 left handed) and 13 males (all right handed)) participated in this experiment. All participants were undergraduate students recruited via an announcement on Facebook. Participants received 8 euros as compensation for their participation. The ethical committee approved this study and participants received a debriefing after completing a single 40 min session. All participants were naive with respect to the purpose of the experiment.

#### Material, stimuli and procedure

In Experiment 2, we used exactly the same material, stimuli and procedure as in Experiment 1 with the exception that the inducer and the diagnostic tasks were randomly intermixed. For each block, an instruction was given with the written words “IN” or “ALL” to inform participants whether they had to perform the magnitude comparison task only on digits inside the memorized sequence (diagnostic task: go-nogo paradigm) or on all presented digits (inducer task: respond all paradigm), respectively. Half of the participants received these instructions before the encoding phase while the other half of the participants received these instructions after the encoding phase. The entire block was introduced again at the end of the experiment if the participant responded erroneously to the control phase of this block, regardless of the task (inducer or diagnostic tasks).

The response mapping was counterbalanced across participants. Half of the participants started with a SNARC compatible mapping while the other half started with a SNARC incompatible mapping. The response mapping was switched after 20 blocks (10 inducer and 10 diagnostic blocks).

### Result and discussion

#### Inducer task—respond all procedure

**Figure 3:**
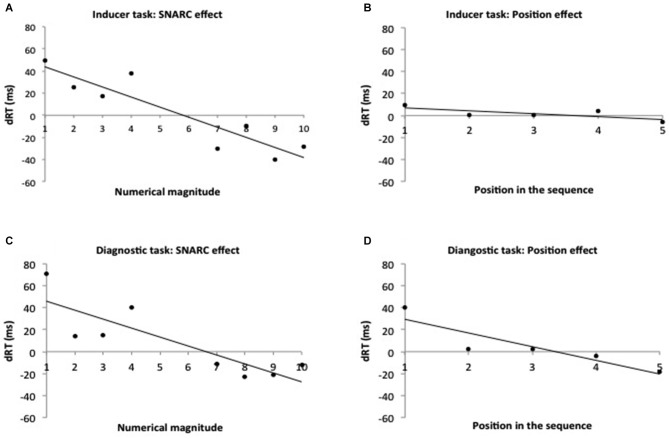
**Experiment 2: the SNARC effect and the ordinal position effect in the inducer and the diagnostic tasks**. Observed data and regression line of Experiment 1, representing RT differences between right and left hand responses in function of the numerical magnitude (in the inducer task **(A)**—in the diagnostic task **(C)** and in function of the ordinal position of digits in working memory (in the inducer task **(B)**—in the diagnostic task **(D)**. Positive values reflect faster left than right responses.

The data of one participant were removed from the analysis because he did not follow the task instructions and did not respond during the classification phase. Another participant was excluded because he made too many errors (more than 2.5 SDs above the mean of errors) during the inducer task. We took into account only blocks with an accurate control phase and correct trials with RTs larger than 250 ms (two data points were discarded with this cut-off). During this task, participants performed correctly on average 11.72 blocks (SD = 2.56) on 10 blocks. During the magnitude comparison task, the average reaction time was 513.51 ms (SD = 72.88 ms) and the average number of errors was 4.70% (SD = 2.63). For the same reason as in the previous experiment, analyses for both tasks (inducer and diagnostic) were performed on all numbers except the numbers 5 and 6. Again, as in Experiment 1, an extra analysis on the entire range of numbers showed the same pattern of results. As in the previous experiment, we investigated whether a SNARC effect was obtained in the inducer task by using a repeated measure ANOVA with numerical magnitude (2: small numbers 1–4, large numbers 7–10) and response side (2: left, right) as within-subjects factors and time of instruction (2: before-after the encoding phase) as the between subject factor. The time of instruction did not interact significantly with the other factors. Only the interaction between numerical magnitude and response side was significant (*F*_(1,48)_ = 17.48, *p* < 0.001, $\eta_{p}^{2}$ = 0.27), indicating the presence of a SNARC effect. Indeed, participants responded faster to small digits with the left-hand side (mean RT = 484.93 ms, SD = 13.26) than with the right-hand side (mean RT = 500.02 ms, SD = 14.16) while they responded faster to large digits with the right-hand side (mean RT = 492.11 ms, SD = 11.16) than with the left-hand side (mean RT = 518.76 ms, SD = 12.03). Further, the presence of the ordinal position effect was investigated for memorized numbers using repeated measures ANOVA with ordinal position (5: from 1 to 5) and response side (2: left, right) as within-subjects factors and time of instruction (2: before-after the encoding phase) as between subjects factor. The interaction between ordinal position and response side, suggesting an ordinal position effect, was not significant (*p* = 0.56). The analyses with the regression approach confirmed the same results. Concerning the SNARC effect, the regression slope differed from zero (*t*_(49)_ = −4.43, *p* < 0.001) (Figure 3A) but this was not the case for the ordinal position effect (*t*_(49)_ = −1.32, *p* = 0.19) (Figure 3B). In sum, as in Experiment 1 the inducer task resulted in a SNARC effect but no ordinal position effect.

#### Diagnostic task—go-nogo procedure

During this task, participants made on average a bit more than one error to the control phase across 10 blocks (for both conditions: small-left/large-right and small-right/large-left). As such, participants required on average 11.37 blocks (SD = 1.90) to finish the task having performed 10 correct sequences. During the magnitude comparison task, the average reaction time was 680.11 ms (SD = 90.88 ms) and the average number of errors was 5.72 % (SD = 3.22). Only one data point was discarded with the RT cut-off of 250 ms. Given that participants responded only to digits inside working memory, we used repeated measures ANOVA with numerical magnitude (2: small, 1–4; large, 7–10), ordinal position (5: from 1 to 5) and response side (2: left, right) as within-subjects factors and time of instruction (2: before-after the encoding phase) as the between subject factor. This ANOVA indicated a main effect of position (*F*_(4,192)_ = 12.00, *p* < 0.001, $\eta_{p}^{2}$ = 0.20). Average RTs per position increased gradually (674, 686, 704, 722, 712 for each position, respectively). The linear trends of these RTs was confirmed by the polynomial contrast (*F*_(1,48)_ = 45.66, *p* < 0.001, $\eta_{p}^{2}$ = 0.49), suggesting a serial search strategy. The analysis revealed a significant interaction between ordinal position and response side, reflecting the presence of an ordinal position effect (*F*_(4,192)_ = 5.54, *p* < 0.001, $\eta_{p}^{2}$ = 0.10). Importantly, also the interaction between numerical magnitude and response side (indicative for the SNARC effect) was significant (*F*_(1,48)_ = 6.73, *p* < 0.05, $\eta_{p}^{2}$ = 0.12). The triple interaction between numerical magnitude, ordinal position, and response side was not significant (*F*_(4,192)_ = 1.68, *p* = 0.16, $\eta_{p}^{2}$ = 0.03). These results were confirmed using the regression analysis. The dRTs decreased 12.46 ms per ordinal position (*t*_(49)_ = −3.42, *p* < 0.005) (Figure 3D) and 10.98 ms per numbers (*t*_(49)_ = −3.22, *p* < 0.005) (Figure 3C), indicating the presence of an ordinal position effect and a SNARC effect.

One could ask whether the presence of both effects simultaneously in Experiment 2 was not due to averaging. Indeed, the possibility remains that the position effect was dissociated from the SNARC effect at the individual level but not at the group level. That is, some participants would show the SNARC effect only while other participants would show the ordinal position effect only. If this were the case, we should have observed a negative correlation between the ordinal position effect and the SNARC effect. However, this was not observed (*r* = 0.019, *p* = 0.89).

In sum, during the inducer task, as in Experiment 1, we observed the presence of a SNARC effect. During the diagnostic task, both an ordinal position effect and a SNARC effect were observed. The moment of instruction (before or after the encoding phase) had no influence on either the SNARC effect or the ordinal position effect.

## Discussion

In two experiments we investigated the relation between the SNARC effect and the ordinal position effect. The ordinal position effect is believed to result from the creation of temporary position-space associations. If the SNARC effect also results from the activation of these temporary position-space associations (e.g., van Dijck and Fias, 2011), then it logically follows that the SNARC effect and the ordinal position effect are mutually exclusive. If, on the other hand, the SNARC effect results from the activation of long-term semantic representations and not from the activation of temporary-space associations, then both effects can in principle be observed at the same time (e.g., Ginsburg et al., 2014). Both our experiments consisted of an inducer task and a diagnostic task. The goal of the inducer task was to activate the associations between lateralized responses and the canonical order of digits, resulting in the SNARC effect. In the diagnostic part we measured the influence of pre-activating the SNARC effect on the categorization of a newly memorized sequence of numbers. The inducer task and the diagnostic task were presented blocked-wise (Experiment 1) or randomly intermixed (Experiment 2).

The results of the inducer task were highly similar in both experiments and similar to the results obtained in a previous study (Ginsburg et al., 2014). That is, both in Experiment 1 and in Experiment 2 the diagnostic task resulted in a SNARC effect but no ordinal position effect. The results differed between both experiments on the diagnostic task. In Experiment 1, the inducer task and the diagnostic task were presented blocked-wise. Only an ordinal position effect but no SNARC effect was observed. Additional exploratory analyses on the diagnostic task revealed that the SNARC effect interacted with time. This interaction suggests that the inducer task did have an influence on the presence of the SNARC effect during the diagnostic task, but that this influence was limited in time. We followed up on this hypothesis in Experiment 2 by randomly inter-mixing the inducer and the diagnostic tasks. Intermixing both tasks assured that the activation of the associations resulting in the SNARC effect (inducer part) remained active throughout the entire experiment. Importantly, as a consequence of this manipulation, both the SNARC effect and the ordinal position effect were observed simultaneously.

The current results are consistent with the suggestion that the SNARC effect reflects spatial associations with pre-existing activated bindings whereas the ordinal position effect reflects spatial associations with new temporary bindings needed for the task (Ginsburg et al., 2014). A tentative interpretation of this idea is that, in most everyday life situations, numbers are presented in their canonical order. Activation of this canonical long-term representation would automatically take place when we have to deal with numbers (as in a magnitude comparison task or in a parity judgment task). This activation is a prerequisite to create spatial associations between this canonical order and response side (e.g., the SNARC effect). However, when retrieval of numbers belonging to a new memorized sequence is needed (go-nogo task), an imbalance is established between the activation of the irrelevant canonical order and the activation of the new, non canonical, relevant sequence. Because the retrieval of the new memorized sequence is needed to perform the task (go-nogo procedure), the latter would receive more activation, resulting in an ordinal position effect. In Experiment 1, because the inducer and the diagnostic tasks were performed separately, the activation of the canonical representation of numbers was still lower than the activation of the new memorized sequence during the diagnostic task. As a consequence, the influence of the inducer task was short-lived illustrated by the interaction between the SNARC effect and time during the diagnostic task. In the second experiment, on the other hand, the inducer and diagnostic tasks were randomly intermixed and the switch between tasks requires constant updating of the current task set (canonical order and newly memorized order) (Monsell, 2003). Because of this task switching, bindings between memorized items must be established strong enough to be armed against interference but loosely enough to be rapidly dismantled (Oberauer, 2010). As such, during diagnostic blocks, a competition likely takes place between the activation of the irrelevant sequence of numbers activated in long-term memory (e.g., the canonical order of numbers) and the relevant sequence of numbers (newly memorized sequence of numbers) in working memory that needs to be maintained and retrieved (for a similar reasoning, see Oberauer, 2009; Szmalec et al., 2011). As a result, the ordinal position effect and the SNARC effect were simultaneously observed. A final relevant observation is that the SNARC effect and the ordinal position effect did not interact. This lack of interaction is congruent with the idea, as proposed above, that the SNARC effect and the ordinal position effect reflect the activation of different representations (long-term pre-existing representations and temporary working memory representations, respectively). Firm replication of this observation is however needed as it concerns a reasoning on the basis of the absence of an effect.

Even if the SNARC effect and the ordinal position effect reflect the activation of different types of representations (digits in long-term memory and in working memory, respectively), a similar attention mechanism might underlie both types of representations. Indeed, Fischer et al. (2003) demonstrated that merely looking at numbers can cause a shift of attention to the left or to the right side of space, depending on the canonical position of the digit in the sequence. The presentation of small numbers (1 and 2) induced a spatial shift of attention to the left, while the opposite pattern was observed for large numbers (8 and 9). More recently, van Dijck et al. (2014) demonstrated that such spatial shifts of attention could also be induced by the ordinal position of an item in a newly memorized sequence. This was demonstrated by combining the go-nogo procedure used by van Dijck and Fias (2011) with the spatial attention paradigm of Fischer et al. (2003). Spatial lateralized targets had to be detected only if a centrally presented number (or letter) belonged to a memorized sequence. Spatial shifts of attention were observed in accordance with the position of the item in working memory. A right dot advantage emerged with the retrieval of an element at the end of the memorized sequence. Such observations illustrate the role of spatial attention in number-space associations and add supplementary evidence to the implication of working memory on those associations. Indeed, working memory has been conceptualized as an attention-based process (e.g., Cowan, 1999; Oberauer and Hein, 2012) in which attention is a mechanism allocated for selection (Allport, 1987). Within this perspective, we consider the impact of working memory and attention as the influence of one and the same process.

The idea that the ordinal position in activated long-term memory and in a new memorized sequence can induce spatial shifts of attention does not imply that those representations themselves are spatial in nature. Our results demonstrate that shifts of attention (or alternative causes of the response bias) are more likely to be induced during the retrieval of the item from the sequence during the classification phase. Indeed, in previous work it was demonstrated that the ordinal position effect is not observed if retrieval is not required by the task (Ginsburg et al., 2014). On top of this, we observed in the Experiment 2 that the moment of instruction (before or after encoding) had no influence on the ordinal position effect and the SNARC effect, neither during the diagnostic or the inducer tasks. This suggests that the mapping between response side and the item is created during the retrieval of the target from the sequence to which it belongs (long-term or short-term representations).

In conclusion, the main result of the current study is that the SNARC effect and the ordinal position effect are not mutually exclusive. Therefore, both effects do not seem to be the result of the same underlying representation. Spatial associations can be tied to both activated long-term representations and to temporary short-term representations simultaneously. Furthermore, it was also observed that the time of instruction had no influence on the ordinal position effect. This observation strengthens the initial claim (e.g., Ginsburg et al., 2014) that spatial associations responsible for the ordinal position effect are created during the retrieval of the item from working memory.

## Conflict of interest statement

The authors declare that the research was conducted in the absence of any commercial or financial relationships that could be construed as a potential conflict of interest.
